# 702. Risk Factors for Acute Gastroenteritis Among Patients Hospitalized in 5 Veterans Affairs Medical Centers, 2016–19

**DOI:** 10.1093/ofid/ofab466.899

**Published:** 2021-12-04

**Authors:** Neha Balachandran, Jordan Cates, Anita Kambhampati, Vincent Marconi, Sheldon T Brown, Maria C Rodriguez–Barradas, David Beenhouwer, Mark Holodniy, Cynthia A Lucero-Obusan, Cristina Cardemil, Umesh D Parashar, Sara Mirza

**Affiliations:** 1 Oak Ridge Institute for Science and Education, Oak Ridge, TN, Atlanta, GA; 2 Division of Viral Diseases, National Center for Immunization and Respiratory Diseases, Centers for Disease Control and Prevention, Atlanta, Georgia; 3 IHRC, Inc. contracting agency to the Division of Viral Diseases, Centers for Disease Control and Prevention, Atlanta, GA; 4 Atlanta VA, Atlanta, GA; 5 James J Peters VAMC, Bronx, NY; 6 Michael E. DeBakey VAMC, Houston, TX; 7 VA Greater Los Angeles, Los Angeles, CA; 8 Department of Veterans Affairs, Palo Alto, CA; 9 Division of Viral Diseases, Centers for Disease Control and Prevention, Atlanta, GA

## Abstract

**Background:**

In the United States, an estimated 179 million acute gastroenteritis (AGE) episodes occur each year. Identifying factors contributing to AGE susceptibility and severity is important to address the high disease burden of AGE among adults. The primary objective of this analysis was to identify risk factors for all-cause AGE, norovirus-associated AGE and severe AGE among hospitalized adults.

**Methods:**

We analyzed data from 1029 inpatient AGE cases and 624 non-AGE controls enrolled prospectively from December 1, 2016 – November 30, 2019 from 5 Veterans Affairs Medical Centers (Atlanta, Bronx, Houston, Los Angeles, Palo Alto). Standardized patient interviews and medical chart abstractions were conducted to collect demographics, exposure history, and underlying medical conditions. Stool samples from participants were tested for 22 pathogens using the BioFire Gastrointestinal Panel. Severity of AGE was determined using a 20-point modified Vesikari score (MVS) and severe AGE was defined as a MVS score of ≥ 11. Multivariate logistic regression was performed to assess associations between potential risk factors and outcomes.

**Results:**

Of the total AGE cases, 551 (54%) had severe AGE; 44 (4%) were norovirus positive. Risk factors for all-cause AGE vs. non-AGE controls included household contact with a person with AGE in the past 7 days (aOR=2.9, 95% CI:1.3-6.7), severe renal disease (aOR=3.1, 95% CI:1.8-5.2), human immunodeficiency virus (HIV) (aOR=3.9, 95% CI:1.8-8.5), and immunosuppressive therapy (aOR=5.6, 95% CI:2.7-11.7). Factors associated with norovirus positivity by univariate analysis were contact with a person with AGE outside (OR=4.4, 95% CI:1.6-12.0) and within (OR=5.0, 95% CI:2.2-11.5) the household in the past 7 days. Detection of any viral pathogen (aOR=4.0, 95% CI:1.7-9.5) was a risk factor for severe AGE.

**Conclusion:**

Our findings suggest that inpatients with HIV or severe renal disease, on immunosuppressive therapy, or in contact with a person with AGE within household are at higher risk for all-cause AGE. Patients with these medical conditions should be monitored for AGE related hospitalizations and may benefit from targeted AGE prevention messaging.

**Disclosures:**

**Vincent Marconi, MD**, **Bayer** (Consultant, Scientific Research Study Investigator)**Eli Lilly** (Consultant, Scientific Research Study Investigator)**Gilead Sciences** (Consultant, Scientific Research Study Investigator)**ViiV** (Consultant, Scientific Research Study Investigator)

